# Smart home technology to support engagement in everyday activities while ageing: A focus group study with current and future generations of older adults

**DOI:** 10.1371/journal.pone.0317352

**Published:** 2025-01-28

**Authors:** William Son Galanza, Jens Offerman, Sofi Fristedt, Susanne Iwarsson, Nebojsa Malesevic, Steven M. Schmidt

**Affiliations:** 1 Faculty of Medicine, Department of Health Sciences, Lund University, Lund, Sweden; 2 School of Health and Welfare, Jönköping University, Jönköping, Sweden; 3 Faculty of Engineering, Department of Biomedical Engineering, Lund University, Lund, Sweden; All India Institute of Medical Sciences - Patna, INDIA

## Abstract

Despite the potential of smart home technologies (SHT) to support everyday activities, the implementation rate of such technology in the homes of older adults remains low. The overall aim of this study was to explore factors involved in the decision-making process in adopting SHT among current and future generations of older adults. We also aimed to identify and understand barriers and facilitators that can better support older adults’ engagement in everyday activities. Focus group discussions were used to explore the perspectives of people from diverse age groups (30–39, 50–59, and 70-79-year-olds). Three focus groups met twice at a lab designed as a two-room home equipped with SHT. Our findings revealed that the participants’ decision-making process for adopting SHT involved designs that must be adapted to the changing physical abilities and diverse needs of users. Some conditions, such as ideas for re-invention, were identified after the integration of SHT. Concerns about reliability, complicated interfaces, and value to the user influenced the decision to adopt SHT, highlighting the importance of these factors for successful implementation. Some participants did not fully understand what SHT is nor perceive its benefits, but they expressed a desire to acquire the skills and knowledge to operate SHT. Furthermore, participants desired SHT that can support an active lifestyle. The perceived advantages of SHT include enhancing the sense of security and safety, which can facilitate engagement in everyday activity. Some participants experienced a positive impact on quality of life, related to comfortable living with the implementation of SHT. Adults across age groups perceive that SHT can enhance engagement in everyday activity and the sense of safety and security. However, it is essential to identify solutions for better usability. More collaborative efforts involving diverse stakeholders are vital to bridge the disconnect between SHT design and users’ needs and preferences.

## Introduction

Active ageing refers to engagement in everyday activities that enhance older adults’ quality of life and well-being, leading to improved cognitive and physical functioning, extended lifespan, and enhanced independence [1,2]. One of the practical recommendations to support active ageing is implementing smart home technology (SHT) [3,4]. The benefits of using technology to support active ageing among older adults have been extensively advocated for [5–8], but the uptake of SHT among older adults remains low. To incentivise and enable current and future generations of older adults to remain active and independent [4,9,10], more knowledge about the challenges and benefits of SHT adoption and use is warranted.

Smart home (SH) refers to homes equipped with a combination of SHTs that are integrated into intelligent systems to provide people with, for instance, comfort and safety through controlling the home environment [11–13]. SHT can include video monitors, motion sensors, security and health related alarms, smart planners, voice commands, and thermostats [14,15]. SHT has been available since the 1980s [16] and said to positively impact people’s economic, social, health-related, security, and emotional aspects of life [4]. However, there is a gap in skills and knowledge about SHT among the ageing population, and the adoption of SHT is far from mainstream [17]. While independence and safety are the main reasons for using and choosing technology [18], that is, reasons in line with SHT designers’ intentions, as reported in recent studies, SH is not perceived to support active ageing [18,19]. Thus, many older adults hesitate to adopt and use SHT [20]. Low rates of use and lack of knowledge about the advantages of SHT highlight the need to advance research to understand the decision-making process for the adoption and use of SHT.

According to the diffusion of innovation theory (DOI) [21], adopting an innovation (for instance, SHT) is an ongoing process. The innovation-decision process is a series of steps that a person goes through to adopt or reject an innovation. It starts with becoming aware of the innovation, forming an attitude, deciding whether to adopt or reject it, implementing the new idea, and finally confirming the decision. In other words, a series of choices and actions need to be considered, such as finding out whether SHT can support a person to meet his/her needs, desires, and interests. This leads to evaluating the new idea and deciding whether to adopt the innovation into daily life [21]. Looking closer at the innovation-decision process can provide a structured approach to understand and address the factors influencing the adoption of SHT.

Although the rate of technology adoption among older adults is increasing [22], age-related factors such as coping, social support and characteristics of older adults need to be considered as these factors may influence technology adoption [14,23,24]. Exploring factors influencing adoption, attitudes toward, desires and needs toward SHT is essential to understand how to reduce age-related digital disparities [25]. However, there is a lack of research that examines the adoption of SHT from a generational perspective, also considering the potential diversity among current and future generations of older adults. Research shows that attitudes and preferences for SHT sometimes differ and sometimes align across generations with individual differences within generations [18,26]. Recognising the diversity and non-diversity from generational perspectives is essential when exploring SHT adoption perspectives with a wider range of users. For example, lower SHT adoption rates might be more related to individual attitudes and experiences with SHT and not merely generational. Furthermore, Chang and Nam [27] argued that there is a considerable need for research on SHT that can support independent living, such as safety and convenience, among all age groups.

Previous research on SHT has mainly focused on technological, infrastructure, and architectural aspects. Consequently, empirical knowledge is scarce on perceptions and viewpoints regarding the benefits and challenges of using SHT to support engagement in everyday activity [3,4,10]. This highlights the need for research describing attitudes towards, needs for, and adoption of SHT to support engagement in everyday activities from the perspective of current and future generations of older adults. The overall aim of this study was to explore factors involved in the decision-making process in adopting SHT among current and future generations of older adults. We also aimed to identify and understand barriers and facilitators that can better support older adults’ engagement in everyday activities at home as they age.

## Materials and methods

In this qualitative study we used focus group discussions to explore participants´ perspectives and experiences [28]. Focus group discussions were chosen as they allow in-depth, interactive discussions among participants and could uncover common, diverse and complex needs and desires for SHT. Moreover, the iterative discussions and permissive group environment in the focus groups provide an opportunity to capture similarities and contrasting attitudes and experiences [28].

The Swedish Ethical Review Authority approved the study (No: 2023-00119-01).

### Participants and recruitment

Participants were recruited by sending invitations to existing mailing lists (including people who had expressed interest in participating in research and other activities), the network of the User Board at the Centre for Ageing and Supportive Environments (CASE) at Lund University, and the researchers’ networks. The recruitment period started from 2023-04-01 to 2023-05-30. A leaflet was distributed in public areas, such as universities, hospitals, receptions of training facilities, supermarkets, cafeterias, and libraries, to recruit participants from younger age groups. People who expressed interest via mail or telephone received an information letter, were contacted by telephone or mail, and were informed about the purpose and implementation of the study. Participants who were available to participate on the planned dates for the focus group sessions were finally included.

Focus groups offer good reflection and involvement among participants [29]. To collect a variety of perspectives surrounding SHT and increase confidence in capturing patterns of perspective and adoption of SHT [30], we organised three focus groups with two sessions each (i.e., six sessions in all). The time between each focus group session allowed participants to reflect more critically on their attitudes towards SHT between sessions, which generated more contextually grounded data.

In total, 15 people participated (Table 1). Before the first sessions, all participants were encouraged to raise concerns (none were expressed) and questions about the study. They signed informed consent, including consent for audio and video recordings.

**Table 1:** Participant characteristics and distribution in three focus groups (N = 15).

Demographic variable	Focus group 1 (n = 6)	Focus group 2 (n = 5)	Focus group 3 (n = 5)	Total
**Age, years**				
30–39	0	1	2	3
50–59	1 ^a^	0	3 ^a^	4
70–79	5	4	0	9
**Gender**				
men	3 ^a^	2	2 ^a^	7
women	3	3	3	9
**Education**				
elementary			1	1
high school	1 ^a^	2	3 ^a^	6
university	5	3	1	9
**Civil Status**				
single	3 ^a^	3	3 ^a^	9
partnered	3	2	2	7
**Income (SEK)**				
<20,000–29,000	1	3	3	7
30,000–39,000	5 ^a^	2	2 ^a^	9
**Health Status**				
good	0	2	1	3
excellent	6 ^a^	3	4 ^a^	13
**SHT Experience**				
yes	5 ^a^	0	2 ^a^	7
no	1	5	3	9

SHT, smart home technology; SEK, Swedish crown.

^a^ One participant took part in one session of Focus Group 1 and two sessions in Focus Group 3 due to scheduling constraints and unavailability to attend both sessions in Focus Group 1.

### Procedure

We developed a set of open-ended questions [29,30] focused on topics relevant to the use of SHT (i.e., attitudes, desires, needs, barriers, and facilitators to adoption of SHT) (Table 2). The content of the questioning route was built on results of studies that broadly explored different types of technologies in several generations [18,26] and was utilised to stimulate individual perspectives and experiences in the discussion while maintaining focus on the study aim.

**Table 2:** Example of the open-ended questions used during the focus group discussions.

Session 1	Session 2
What is your experience with SHT? Which SHT are you familiar with? Have you seen it in stores, television, online, advertising, or elsewhere?	What matters to you if/when choosing, adopting and starting to use SHT? Is design, function, sustainability, economy, integrity, safety, and health important? If so, how and why?
Does SHT support your everyday activities? If so, how?	Is there anything stopping you from using SHT? If so, what?
Are you aware of any SHT available on the market that would suit the needs and desires we have discussed today?	How do you expect (as you get older) that SHT will affect you? How will it affect your everyday activities, social life, security participation or health? How will it affect health or social care services? Other effects, if so what, how or why?

SHT, smart home technology.

The focus groups were held at the Movement and Reality Lab (MoRe-Lab) at the Faculty of Medicine, Lund University—About MoRe-Lab | MoRe-Lab (lu.se). This state-of-the-art facility for experimental health sciences features a reality lab, essentially an apartment, designed as an instrumented home environment platform. The lab allows researchers to study complex interactions between people, technology, behaviour, cognition, and activity within a home setting. The apartment was equipped with a selection of technologies representing a standard combination of SHT to support comfort, security, safety, and economic gains through efficiency [11]. We focused on SHT, which had been highlighted by current and future older adults in previous studies [18,26]. The selection of SHT installed at the MoRe-Lab included commonly available SHT that we considered applicable across different generations (Table 3).

**Table 3:** Smart home technology presented at the MoRe-Lab during the focus group sessions.

Smart Home Technology
Door Contact Input Infrared Camera Detector Robot Vacuum Cleaner Security Keypad Smart Bulb Smart Coffee Maker Smart Home Hub Smart Plug Video Door Phone Water Detector

The first session started with an interaction among participants on their understanding of and previous experience with SHT. A presentation of the SHT available in the MoRe-Lab and a short video (NookBox—Smart Home Security—YouTube) were presented by moderators to stimulate the discussion. Using the questioning route, the participants discussed their attitudes, desires, and needs towards SHT to support continued engagement in everyday activities while ageing. The second session started with a summary of the previous session. Using the questioning route, discussions focused on the participants’ perspectives on facilitators and barriers to SHT use. The discussions included prioritised technology and functions to generate knowledge about what technologies and functions support engagement in everyday activities at home. The focus groups were conducted and facilitated by two PhD students (JO, moderator; WSG assistant moderator), with one senior researcher (SF) moderating the first two sessions and another (SMS) observing and recording from a control room. Notes were taken by the assistant moderator, which afterwards helped the moderators reflect on the depth of discussions and prepare for the next session. Each session lasted on average, 90 min.

### Data analysis

The audio recordings were transcribed verbatim by WSG (sessions 1,3,5) and JO (sessions 2,4,6), which provided an accurate record and deep understanding of the conversations. The video recordings supported the transcription to identify the group participants, link to their narratives correctly, and observe the group dynamics. WSG and JO initially analysed the transcripts following a theory-driven deductive thematic approach [31,32]. This approach enabled the analysis process to focus on predefined themes and sub-themes while remaining open to emerging insights from the data. The framework used not only enhanced the rigour of the analysis but also provided a structured framework for interpreting the complexities of different stages in SHT adoption. An analytic framework based on the five-step decision-making process derived from the DOI theory [21], were used to develop and define themes by WSG and JO with the support of SMS and SF (Table 4). We continued to develop the themes and sub-themes as the analysis progressed. According to the DOI theory, an innovation is a new product that provides advantages to a group or market [21]. The entry of the product into the market initiates the diffusion of the innovation, which can occur through communication. For this study, innovation refers to the emergence of SHT, and diffusion of innovation refers to the five-step decision-making process.

**Table 4:** Themes and sub-themes analytic framework used for the deductive analysis.

Five-stage decision-making process	Theme	Sub-theme
***Stage 1*. *Awareness/Knowledge***	Awareness and knowledge of SHT.	Own desire to understand SHT
		Increased awareness supported by politicians and change agents
***Stage 2*. *Persuasion***	Desired, non-desired and needs of SHT	Perceived advantages of SHT
		The potential impact of SHT on users’ functioning and health
		User data integrity with SHT implementation
		Perceived need for SHT
***Stage 3*. *Decision***	Determining ease of use through trial	
***Stage 4*. *Implementation***	Integration of SHT into the home environment	Dealing with uncertainty and consequences of implementation
		Ideas for re-invention that would support practical implementation
		Ideas for re-invention for AI-supported SHT
***Stage 5*. *Confirmation***	Positive reinforcement or rejection of the adopted SHT	Experiences supporting adoption.
		Reject SHT after adoption.

SHT, smart home technology.

Note. Developed based on the Diffusion of Innovation Theory [21].

Next, one of the researchers (WSG) immersed himself in the transcripts to generate initial codes, and annotations were made to develop an in-depth understanding of the participant’s attitudes, desires, needs, facilitators, and barriers to adopting SHT. Codes were then assigned to Rogers’ [21] five-step decision-making process (knowledge, persuasion, decision, implementation, and confirmation). This was followed by a continuous discussion among all co-authors of the emerging codes, sub-themes, and themes. WSG performed a second round of coding independent of the first set of codes to validate the coherence of themes and sub-themes to the codes and their transcript origin. This clarified similarities and differences of the codes that overlap across the five-step decision-making process. Analyses were aided by the NVivo software tool [33,34].

## Findings

The participants acknowledged that their experiences with SHT were limited, with most having little or no direct interaction or usage history. Focusing on decision-making and adoption of SHT five themes emerged from the analysis (Table 4). The five themes, which represent each stage in the five-stage decision-making process of the DOI, illustrate both individual and collective perspectives on SHT adoption. The awareness and knowledge of SHT, the initial stage in the adoption process, were driven by users’ desire to better understand SHT and change agents. In the persuasion stage, participants identified some aspects and formed attitudes that would persuade or not persuade them to adopt SHT. Determining the ease of use through trials that occurred at the decision stage helped address uncertainties and refine usability, leading to a decision to adopt or reject SHT. Some of the participants who proceeded to the implementation stage successfully integrated SHT into their homes, requiring innovative approaches and re-invention of SHT. At the confirmation stage, the participants experienced positive reinforcement or rejection of the adopted SHT. Overall, positive experiences, like increased safety and convenience, promote adoption, while concern for privacy and data security can lead to rejection.

### Awareness and knowledge of SHT

The participants displayed different levels of knowledge and experience in SHT. Poor knowledge was primarily related to low exposure to SHT, which the participants considered compromising their adoption of such technologies.

#### Own desire to understand SHT

Despite some participants’ initiatives to enhance their knowledge about SHT through digital platforms, there was still poor recognition of its meaning and the types of SHTs available on the market to support engagement in everyday activities.

A strong desire to enhance the level of knowledge of which SHTs can address current and future needs was repeatedly raised by the 50–59 and 70–79 year olds, but not by the 30–39 year olds.

*“That day, you lose your abilities. That is when it is essential to know what SHT is available. I checked this AI chat for proactive SHT support, such as the smartwatch. I want to be at the forefront. I want to be thinking about the future.”*

*(F13*, *70–79)*.

#### Increased awareness supported by politicians and change agents

The role of politicians was regarded as necessary, both in setting efforts to enhance awareness of SHT use in and out of the home and in providing guidelines that consider the skills of potential users when implementing SHT.

*“Our discussion must be shared with society so that politicians and others can hear about it and make decisions about this sort of thing*.”

*(F13*, *70–79)*.

Participants viewed change agents, such as SHT professionals, family, or friends, as facilitators for adopting SHT.

*“There is so much we do not know. So, I want someone to tell me what I need. We need someone to show us how we can get help from SHT and make it easier for us.”*

*(F12*, *70–79)*.

For some of the participants, family and friends were considered to influence them the most: “*Friends trigger my interest*. *As a result*, *I buy such technology because others have mentioned it to me*.*” (M7*, *50–59)*.

### Desired, non-desired and needs of SHT

Participants’ attitudes, desires, and needs toward SHT were mixed. Both sceptical and positive attitudes were observed that could or could not persuade SHT adoption across the age groups. Concern towards some SHT was observed as participants spoke about potential barriers such as impact on health and invasion of users’ integrity. Such barriers seemed to contribute to a more cautious attitude regarding the potential adoption and successful implementation of SHT in their homes.

#### Perceived advantages of SHT

SHT was primarily considered to save time. The timesaving perspective related to SHT use was considered essential and observed among all participants.

*“We buy time by using SHT. It creates increased flexibility in everyday life that allows me to feel safe and free up half an hour for other things compared to what I would otherwise.”*

*(M4*, *70–79)*.

A positive attitude was also expressed through interest in artificial intelligence (AI), automation, and the potential economic gain through efficient home energy consumption.

*“I like AI because there is a lot that you can do with it. Moreover, I like cameras in the home and a little robot that can provide instruction and reminders about everyday activities.”*

*(F2*, *70–79)*.

One of the main factors the participants thought would persuade them to adopt SHT was the perceived benefits. The benefits of SHT are not only for a single user but extend to other family members, especially those with children living at home. Changes in family status, such as having children and home settings, such as apartments or villas, were seen as influential regarding the will to equip the home with SHT. Some participants were interested in SHT as a potential support for independence and ageing in place. They considered that SHT could keep them safe and secure while they enjoyed engagement in everyday activities:

*“The important thing is that you should be able to live at home for as long as possible, but it has to do with safety.”*

*(F3*, *70–79)*.

#### The potential impact of SHT on users’ functioning and health

It was perceived that the full implementation of SHT can lead to users being confined inside the homes and becoming lonely. Negative emotions arose from not feeling needed to manage the household when automated SHT was installed. Receiving regular reminders from AI systems and saving passwords on applications to control SHT were linked to potential cognitive under-stimulation: *“If you assign all these responsibilities to SHT reminders*, *you will probably neglect the importance of keeping the brain healthy*.*” (F9*, *30–39)*.

It was also mentioned that SHT could potentially minimise activity engagement through automation and manipulation of devices through a remote control or digital app. Examples of activities minimised included the few steps from the living room to the light switch, a robot vacuum cleaner, and a robot grass cutter for outdoor spaces. Some participants counter-argued that the type of activity being considered would not impact its importance on physical health. Turning the light on through the smart hub was acceptable, as minimal physical activity was taken away. In addition, some participants considered using SHTs to save physical strength. Devices with voice commands were mentioned as having the potential to support everyday activities better. However, such functions are unreliable and need to be further developed.

SHT that can support engagement in an active lifestyle was also mentioned. Some focused on physical exercises that could be done through a smart TV, as they could watch a person and learn how to do exercises correctly rather than simply listening. Others focused on SHT, which can connect music to their activities, such as active movement and dancing or rehabilitation movement.

*“Some music is great because we like to move and be active. Also, when you sit with an injured foot and cannot move, you can try to dance or move through SHT.” (M8, 50–59). “You can bring up recommended exercises and do them.”*

(M*7*, *50–59)*.

#### Potential impact of SHT on users’ economy

The cost was another disadvantage of SHT mentioned. However, some participants stated they would accept the cost if the product would be durable. As technology evolves, it can be challenging for potential users to operate new SHTs. Therefore, there was a desire for free technical support and installation from the SHT provider. Similarly, there was a desire for SHT to be cost-effective and allow users to prepare food quickly without using excess gas or electricity. However, some participants expressed the likelihood of investing in sustainable SHT for a longer lifetime.

#### User data integrity with SHT implementation

Concerns about users’ integrity can negatively influence persuasion to adopt SHT. The participants discussed the safety of AI-generated devices and intelligent robots at home from different perspectives. For example, some participants questioned how much autonomy AI robots can develop based on the data they collect from the user, while other participants entirely accepted such devices and were convinced of the robot’s potential support.

*“We do not know the limit. How much can these robots change through the information they have gained? Are we confident that no one inside manipulates it?” (M4, 70–79). “I feel terrified about the idea of having robots at home helping me.”*

*(F1*, *70–79)*.

The 70–79 year olds expressed worry about how AI in SHT processes their data and how that would affect their privacy or bring risk for fraud crimes, but the 30–39 and 50–59 year olds had fewer immediate concerns about such issues.

#### Perceived need for SHT

The perceived need for SHT to support engagement in everyday activity varied among participants based on individual preferences, values, and priorities. The need for SHT for safety and security relates to the potential of SHT to support autonomy and independence. It was also mentioned that there is an increased need for safety and security parallel to increasing age. Some of the SHTs that were mentioned include monitoring sensors, house alarms, cameras, voice commands, and innovative kitchen design.

*“Monitoring all home spaces with voice control to send signals to someone if needed. It is not optimal but having it on would help in 50–75% of those situations.”*

*(M4*, *70–79)*.

Regarding the SHT that can enhance the social isolation and loneliness aspect of day-to-day life, a dog robot and hologram for telephone calls were desired.

*“It is very cool (M8, 50–59 & F6, 50–59: agree). You call someone, and then suddenly, you are sitting here and one with three dimensions.” (M7, 50–59). “It would help with social loneliness.” (F9, 30–39). “I can call and see their faces nearby. We live with SHT today, and I want to learn and develop more.”*

*(F6*, *50–59)*.

The 70–79 year old participants expressed an immediate need for SHT to support autonomy and independent living, while the 50–59 and 30–39 year olds viewed SHT with a more forward-looking perspective. Some participants mentioned that they potentially need SHT for future use once they reach a certain age. Family situations also fuel the need for SHT security systems.

*“When I am 90 years old, I would like to have a camera outside, for example, to see what is happening, especially when I feel like I cannot defend myself.”*

*(F9*, *30–39)*.

Some participants were less concerned about SHT, which is not adapted to age. In contrast, others mentioned user-friendly devices like easy-to-click buttons and screens. On the other hand, concern was raised about the need for SHT to be dynamic and adaptable to the user’s changing health. For example, a complete home installation with SHT at one time point may lose its benefits years later related to the potential decline of the user’s hearing, balance, or other health aspects.

“*There must be a time perspective in relation to the phasing out of eyes, ears, cognition, and memory and that it is getting worse and worse. You must consider the potential declining health of the user if you equip a home for the ageing user.”*

*(F3*, *70–79)*.

Sentiments of forced persuasion to use SHT were also raised. Some of the participants expressed not fully accepting the use of SHT but ended up using it anyway. One reason was for monitoring and safety. Another was social inclusion, the sense of not being part of a digital society and technological advancement without such technology.

*“My daughter forced this smartwatch on me, which is ridiculous. I hate that it can make me feel like I have a problem with my fingers, so I prefer the ordinary watch. I am full of devices at home, like TVs, screens, and gadgets, and you should have those different little things connected. However, I am forced to use them; I do not want to feel left out.”*

*(F11*, *70–79)*.

## Determining ease of use through trial

Participants questioned some practical aspects in the implementation stage, such as complicated functions, reliability, and issues in programming and installation. However, it was noted that this might become less of an issue once they overcome the installation and programming stage.

*“It partly works and partly does not. You need to go through many detours to reach the goal. I do not think technology is adopted. It will develop more and more, but I do not think it feels ready today.”*

*(F9, 30–39)*.

“*But when everything is up and running, it works great and is okay. However, the beginning should be more uncomplicated, specifically the setup and installation, so people do not lose interest when you do not get it right*.”

*(M7*, *50–59)*.

Despite the issues they experienced, some participants wanted to implement SHT without delay, while others were more likely to consider using it in the future.

### Integration of SHT into the home

Implementing SHT into the home environment brought several uncertainties, including the need for further development or re-invention, which must be addressed to ensure successful adoption and support engagement in everyday activity.

#### Dealing with uncertainty and consequences of implementation

The SHT professionals are knowledgeable about the available SHT and can provide support when potential users have trouble installing or operating it. Participants stated that they would need support from an expert on SHT when they have trouble programming or technical issues with their SHT. Also, this support should not be of any cost. When asked who would cover the services, the role of politicians and policymakers was brought into the discussion. In addition, SHT producers and those that market SHT should offer such support for their customers.

Although considered problematic and negatively affecting their adoption of SHT, many of the participants stated that they would solve issues by themselves. This would be done using manuals, trial and error, or the internet, but they would also consider asking relatives for help. Nevertheless, the feeling of not having enough skills still exists if unsuccessful in operating the SHT.

While SHT was considered to promote simple living, our participants expressed that sometimes it did the opposite. Voice commands through SHT can be inconvenient when the intelligent system does not understand the user’s command correctly. There were situations in which the intelligent system was vulnerable and disconnected, complicating the situation even more. One participant felt helpless and did not know what to do when such a situation arose.

*“It felt too complicated to understand. Sometimes, it starts talking out of nowhere. The app to control lights and curtains took an entire day to complete, so it was not easy with this SHT. When it does not work, it is complex and not functional.”*

*(M7*, *50–59)*.

#### Ideas for re-invention to support practical implementation

SHT needs to be developed further or re-invented to effectively respond to the situation, benefit users, better support independence, and provide an enjoyable experience when using such devices. Participants preferred simple designs and standard symbols that facilitate familiarity. The luxurious design was unpopular among the group, and they preferred SHT with only essential functions that could be personalised. External designs that are not user-friendly are wished to be re-invented, such as combining digital and manual functions, an easy-to-click button, and fewer cables. It was also suggested that all different mobile applications controlling SHT be consolidated into one application.

*“I prefer a SHT device that is easy to understand, does not keep updating itself, and changes too much. So, if buttons change or change places, it can cause problems. There should be some universal solution to the symbols.”*

*(F9*, *30–39)*.

Scepticism was expressed about SHT’s reliability. Limited reliability was attributed to its efficiency and the need for further development. One participant experienced an issue with home cameras not capturing the faces of thieves breaking into the house and cleaning devices not covering the intended area.

In addition, the word “smart” in SHT was criticised as not being correct; instead calling it “secured home” was more suitable. Participants reported that some of the current SHTs are not smart; thus, they should not be called smart.

*“I am ambivalent about interpreting the word smart home; it should be secured homes instead. Because of the simplicity and ease it offers. I perceive it wiser, which gives a much deeper meaning than smart.”*

*(M4*, *70–79)*.

Another key desire for re-invention is the monitoring function of SHT. Intelligent monitoring, such as water and electricity consumption, will help to better understand daily consumption. Monitor household activity concerning the impact on the climate, such as food waste, garbage, water, and electricity. Comparing and competing with personal consumption patterns was reported to be fun: *“We would benefit from SHT*, *which would allow you to see your climate benefits and effects*.*” (M4*, *70–79)*.

#### Re-invention ideas for AI-supported SHT

There was also a desire for AI-driven technology to support everyday activity in terms of food preparation and physical activity and monitor vital parameters better. An intelligent wheelchair with auto drive was mentioned to enhance independence. Medicine robots and an effective intelligent system that could respond in cases of emergency were also noted. Another suggestion was SHT that sends signals for physical exercises, or even devices that can remind about exercise based on exercise patterns, especially if the user becomes more sedentary. Refrigerators with built-in features to suggest healthy food recipes based on what is available inside and send a notification on items missing based on users’ dietary habits were also suggested.

*“An app should be connected to the fridge, so you know what to do with everything, such as providing a recipe based on what is in the refrigerator.”*

*(M15*, *70–79)*.

Other participants focused on intelligent features or AI-supported functionality, such as analytics on what training users need to promote health. Also, a notification was suggested to remind the user when and what training should be done next based on the user’s activity pattern.

*“Such an analysis tool or system could, for example, provide tips when electricity is cheaper or tips based on my routine and pattern, such as the activity I do and its potential effects on my health.”*

*(F9*, *30–39)*.

Some participants also expressed an ambivalent attitude towards intelligent notifications. Smart notifications and AI-driven analytics were believed to potentially harm health through stress. For example, notifications and faulty information about doing too much exercise or too little sleep are seen as intrusive to the user’s feelings. Therefore, the user must also be able to personalise such features without further complications.

*“Yes, it is psychological. If it tells you something is wrong, but you feel that you have slept very well, for example, it suddenly makes you stressed, and you do not dare trust yourself.”*

*(M7*, *50–59)*.

### Positive reinforcement or rejection of the adopted SHT

Some participants expressed enhanced comfort, security, and safety, which confirms SHT adoption. The choices to discontinue and reject previously implemented SHT were strongly related to privacy and user benefit.

#### Experiences supporting adoption

Participants who had experienced and installed various SHTs expressed a positive impact on quality of life, having more comfortable living than before. Automated devices and SHT that support chores and keep the house clean were considered to make day-to-day life easier. The 50–59 year olds were more interested in SHT that enhance comfort through ambience with intelligent lights and clean homes than other age groups, especially when arriving home from work. Digital control was valued for comfort at home, such as cooking, making coffee, light control, and curtains. Participants appreciated that they could control their home in the comfort of their couch, bed, or even away from home. Thus, enhancing the feeling of security and safety.

*“Well, it is fantastic. We have such a system at home, and it is excellent. We can control and see everything: alarms, fire protection, alarms on the door, and who is outside. It makes me feel safe and secure. I can see what happens at home when I am not there.”*

*(F6*, *50–59)*.

Despite the discussed needs for improvements, participants from all age groups expressed appreciation for the advancement and development of SHT.

*“I proudly say this creates a freedom I would not have otherwise. How good that you have those functions at home so I can continue to live in my home, even if it is a tenancy; it can decrease the level of anxiety and increase my quality of life.”*

*(M4*, *70–79)*.

#### Rejection of SHT after adoption

Participants experienced little trust in data protection from monitoring cameras. However, sharing health parameter data with healthcare professionals, for example, through distant care, was not considered an issue: “*We did not trust the security of the data collected from the monitoring camera*, *so we took it down and never used it again*.*” (M7*, *50–59)*.

Another factor contributing to rejection was issues following implementation that counteract the ease of use. Such issues can include complications within the home environment, such as cables and furniture, which can hinder the full function of SHT to support engagement in everyday activity. Also, participants said curiosity about SHT faded over time. This could mean curiosity-driven interest in implementing or adopting SHT can lead to rejection rather than long-term implementation.

*“I sold my robot vacuum cleaner instead. It was good, but it hooked up everywhere and became completely unnecessary. Also, some SHTs end up unused in the corner. I think it can be related to curiosity in the beginning. It is fun to play with it initially, but then you get tired of it.”*

*(M15*, *70–79)*.

## Discussion

The exploration of the five stages of SHT adoption in the present study reveals a complex decision-making process that underlines the potential and limitations of SHT adoption among current and future generations of older adults. Our findings contribute to the knowledge of which avenue of SHT implementation can better support user engagement in everyday activities. Moreover, the perceived need for SHT that can support engagement in everyday activity varies among individuals in different age groups, highlighting an intra- and intergenerational variation.

The findings indicate that SHT can enhance perceived safety and security if designed to address users’ needs and desires based on reflecting different stages in life priorities and human needs rather than simply following technological advancements. Thus, it offers users opportunities to participate in everyday activities, as safety and security are prerequisites. Consistent with prior studies, such as those by Oyibo et al. [35] and Arthanat et al. [36], our results confirm that while current and future generations of older adults are generally open to adopting technology that enhances safety and independence, there remains a significant barrier in terms of perceived usability and the personal relevance of such technologies. Moreover, our study adds to the existing literature by demonstrating that the five-stage decision-making process surrounding SHT adoption is not only influenced by practical considerations such as functionality and ease of use but also by more profound, more personal concerns shared by all generations related to autonomy, privacy, and the desire to maintain human control over daily life.

Timesaving was an important facilitator for SHT adoption, valued across all generations in our study. This suggests that even older adults, who might have more free time than younger people, appreciate the efficiency and flexibility that SHT can bring. This cross-generational similarity supports SHT’s potential to support various lifestyles, enhancing its overall marketability and positive influences on quality of life through enhanced engagement in everyday activities. A significant observation from our research is the role of external support and education in adopting SHT. Participants who received guidance and support showed a higher likelihood of embracing SHT to support engagement in everyday activity. This finding suggests a critical role for ongoing education and support structures to assist users and potential users in navigating the complexities of SHT solutions, echoing the sentiments of Lee and Maher [10] and Corbett et al. [37], who noted the importance of initial engagement experiences in SHT adoption.

Our findings reveal high interest in SHTs with intelligent analytics and AI-driven functions. However, these functions are also seen as underdeveloped in terms of user and personalisation opportunities. Similar to a study conducted by Street et al. [38], participants demonstrated cautious and conditional acceptance of SHT. Such findings can be used to communicate the perspective of current and future generations of older adults to policymakers, technology professionals (e.g., product/service providers), and researchers directly involved in the design of features of new SHT [39]. Again, the implication of our findings on the design of SHT, for instance, the voice assistant feature, highlights a potential solution for more acceptable devices among all generations [40,41].

Integrated SHT systems such as automated lighting, voice command controls, and security systems are designed to improve everyday life convenience [11–13]. These systems, if further developed to the needs and desires of older adults, may address age-related challenges through smart automation of doors and windows, smart robotics and interactive activity platforms, making SHT systems an important support in maintaining independence [6]. Our findings show that the oldest generation preferred practical SHT for safety and security rather than technologies for luxury and pure entertainment. For example, a voice-activated system that can control the home environment or request emergency assistance would have a greater impact on an older adult’s quality of life.

While not the focus of the study, some of our findings reveal contrasting perspectives among generations, particularly in relation to safety and security. For older generations, the need for SHT lies in its immediate practicality, especially around autonomy and independent living. In contrast, younger generations seem to view SHT with a more forward-looking perspective. The urgency of adopting SHT for the older generation could be attributed to increased health-related concerns, a desire to reduce dependence on caregivers, or avoid moving into residential care facilities [6]. The younger generations may not feel the same level of urgency for SHT but recognise their potential future value.

We found out that there is a need to develop SHTs that have user-friendly interfaces and simple designs to better support everyday activities. Efforts to mitigate such issues have been reported. Ghorayeb, Comber, and Gooberman-Hill [42], together with some stakeholders, co-designed a user-friendly prototype for data visualisation and the ways information is displayed to the user. They found it essential to receive social and cognitive stimulations and dietary recommendations or activities based on their current state of well-being. In addition, there is a proposal for an offline home automation system that can benefit some settings with internet connection challenges and user cyber security issues. As internet and cloud services are unnecessary for an offline system, they can link the devices while protecting against internet-related crimes [43]. Such study methodology can potentially address privacy and user benefit issues, which are vital reasons to discontinue and reject previously implemented SHT identified in our study.

The participants were worried about how to deal with problems SHT may cause in their homes, such as technology failure and the unreliability of such devices in practical situations. Therefore, SHT systems must cater to people with less technical knowledge and experience. The insights gained can help facilitate more effective implementation strategies. Developers and designers have a substantial opportunity to align more closely with users’ and potential users’ actual needs and lifestyles [44,45]. Our findings revealed that the re-invention of some SHTs should prioritise flexibility and adaptability, allowing users to customise features to their changing physical and cognitive abilities. Such adaptability enhances the practical utility of SHT and addresses the diverse preferences of current and future generations of older adults, potentially leading to broader acceptance and satisfaction. During the focus groups, SHT were demonstrated to provide a clearer understanding; however, only a few participants had tried such solutions. This limited firsthand experience implied that most impressions were based on observations rather than personal use, highlighting a gap between hypothetical knowledge of SHT benefits and practical application.

Our study also contributes to the literature by exploring perspectives on how change agents, which include family members, friends or professionals with expertise in SHT, influence the adoption of SHT. We found out that the adoption of SHT is sometimes forced on older adults without considering technical skills and their willingness to use such devices. As such, Hong et al. [46] pointed out that the issues older adults may face (such as social isolation and digital alienation) when accepting or interacting with SHT should not be overlooked. This is an important aspect that highlights the need to involve the perspectives of other stakeholders, such as potential users and their significant others [20], SHT professionals, healthcare professionals, designers, and engineers, in collaborative scholarly work. This can lead to products and services that are more likely to be accepted and directly address the specifications and functions of SHT identified as essential to support everyday activity. Without studies like this to explore the rationale of the decision-making process behind the adoption of SHT among current and future generations of older adults, the long-term adoption of such technology may not be optimised.

Lastly, our findings offer insights into the use of DOI theory in the adoption process of SHT. This theory provides a structured framework for understanding that SHT spreads through social systems such as change agents and SHT informative materials [21]. We have identified two critical stages in the long-term adoption of SHT: (1) Potential users form favourable or unfavourable attitudes towards SHT and create the need for it, and (2) Ideas for re-invention that consider users’ diverse interests, which can guide the prediction of adoption rates of SHT and guide intervention strategies. However, the DOI theory tends to oversimplify the adoption process by assuming a linear progression from awareness to adoption, overlooking the complexities of real-world decision-making. Additionally, it places less emphasis on contextual factors such as social norms, changes in roles and interests, and individual preferences, which can significantly influence all stages of the adoption decision process and not only in the first stage, as the theory implies.

### Limitations and strengths

The diversity of the participants in this study provided a broad range of responses, insights, and experiences, leading to a more comprehensive understanding of the adoption pattern of SHT. While we involved adults representing three different generations, we were unable to create a focus group with all three generations present due to dropouts and challenges finding meeting times that fit everyone’s schedule, which limited the intergenerational interaction among participants.

A video showcasing one of the SHT systems was presented to enhance engagement and facilitate a more dynamic discussion. This was complemented by the presentation of SHT available in MoRe-Lab, which provided concrete examples. However, a limitation of this approach is the potential for bias introduced by the pre-selection of SHT and the focus on one system. Other systems and possibilities were discussed during the focus group sessions, and participants had time between sessions to explore other SHT possibilities independently.

## Conclusion

We identified critical factors influencing the adoption of SHT that can support engagement in everyday activities among current and future generations of older adults. Varying degrees of enthusiasm and hesitation of the participants reflect the diverse needs and perceptions towards SHT. While there is a strong interest in SHT to enhance independence and quality of life, significant challenges remain regarding usability, accessibility, and the personal relevance of SHT. Therefore, when re-inventing and designing SHT, it is crucial to understand and consider the perspectives of users but also involve current and future generations of older adults in the process. This is important to influence the decision-making and long-term adoption of SHT in line with the DOI theory.

Because adoption is not linear, SHT solutions need to be adaptable to a diversity of users, accommodating changes in health, lifestyle, and personal preferences over time. Therefore, it is essential that SHT developers and designers move away from one-size-fits-all SHT solutions and instead prioritise customisable options for different life stages. Moreover, recognising the non-linear adoption process implies that support and information provided to users must be continuous rather than limited to the initial phase of SHT adoption. Practical applications of such user support could include possibilities for long-term training and ongoing cost-free technical support, ensuring SHT are used to their full potential as users’ circumstances change.

The implications of our findings extend beyond the immediate context of SHT and challenge developers and policymakers to rethink how SHT is integrated with ageing. The enthusiasm for technological solutions should not overshadow the potential for SHT to contribute to increased isolation or inadvertently reduce physical activity. Future research should investigate the long-term social and health outcomes associated with SHT use. It is essential to balance automation with the need for physical engagement and social interaction, which are vital components of everyday activities.
